# Assessment of tumour proliferation by use of the mitotic activity index, and Ki67 and phosphohistone H3 expression, in early‐stage luminal breast cancer

**DOI:** 10.1111/his.14185

**Published:** 2020-08-30

**Authors:** Julia E C van Steenhoven, Anne Kuijer, Robert Kornegoor, Gijs van Leeuwen, Joost van Gorp, Thijs van Dalen, Paul J van Diest

**Affiliations:** ^1^ Department of Surgery Diakonessenhuis Utrecht Utrecht The Netherlands; ^2^ Department of Pathology University Medical Centre Utrecht Utrecht University Utrecht The Netherlands; ^3^ Department of Surgery St Antonius Hospital Nieuwegein The Netherlands; ^4^ Department of Pathology Rijnstate Hospital Arnhem The Netherlands; ^5^ Department of Pathology St Antonius Hospital Nieuwegein The Netherlands

**Keywords:** breast cancer, Ki67, mitotic activity index, phosphohistone H3, reproducibility

## Abstract

**Aims:**

Phosphohistone H3 (PhH3) has been proposed as a novel proliferation marker in breast cancer. This study compares the interobserver agreement for assessment of the mitotic activity index (MAI), Ki67 expression, and PhH3 in a cohort of oestrogen receptor (ER)‐positive breast cancer patients.

**Methods and results:**

Tumour samples of 159 luminal breast cancer patients were collected. MAI and PhH3 scores were assessed by three breast cancer pathologists. Ki67 scores were assessed separately by two of the three pathologists. PhH3‐positive cells were counted in an area of 2 mm^2^, with a threshold of ≥13 positive cells being used to discriminate between low‐proliferative and high‐proliferative tumours. Ki67 expression was assessed with the global scoring method. Ki67 percentages of <20% were considered to be low. The intraclass correlation coefficient (ICC) and Cohen's *κ* statistics were used to evaluate interobserver agreement. The impact on histological grading of replacing the MAI with PhH3 was assessed. Counting PhH3‐positive cells was highly reproducible among all three observers (ICC of 0.86). The *κ* scores for the categorical PhH3 count (*κ* = 0.78, *κ* = 0.68, and *κ* = 0.80) reflected substantial agreement among all observers, whereas agreement for the MAI (*κ* = 0.38, *κ* = 0.52, and *κ* = 0.26) and Ki67 (*κ* = 0.55) was fair to moderate. When PhH3 was used to determine the histological grade, agreement in grading increased (PhH3, *κ* = 0.52, *κ* = 0.48, and *κ* = 0.52; MAI, *κ* = 0.43, *κ* = 0.35, and *κ* = 0.32), and the proportion of grade III tumours increased (14%, 18%, and 27%).

**Conclusion:**

PhH3 seems to outperform Ki67 and the MAI as a reproducible means to measure tumour proliferation in luminal‐type breast cancer. Variation in the assessment of histological grade might be reduced by using PhH3, but would result in an increase in the proportion of high‐grade cancers.

## Introduction

Histological tumour grade is one of the most robust prognostic factors in breast cancer.^1^, ^2^, ^3^, ^4^, ^5^ The modified Bloom and Richardson (BR) Nottingham grading system, which has been globally incorporated in breast cancer guidelines,^6^ reflects three features, i.e. nuclear polymorphism, tubular formation, and mitotic count, the last of which reflects tumour proliferation. By the assignment of a score to each of these features, tumours are divided into three categories. Category 1 contains the well‐differentiated tumours with an inherently good prognosis, and category 3 contains the poorly differentiated tumours.^1^, ^5^

Assessment of histological grade is applied worldwide, and adds important prognostic information to other clinicopathological features in order to guide systemic treatment decisions. Patients with grade 3 tumours are often candidates for treatment with adjuvant chemotherapy, whereas those with grade I tumours are candidates for less toxic hormonal therapy.^6^ A substantial proportion (30–60%) of patients are diagnosed with grade 2 tumours, and in these patients the indication for adjuvant systemic treatment is less clear. Especially in this category of patients, high interobserver grading variability and institutional inconsistencies have been reported.^7^, ^8^, ^9^

Over time, determination of the roles of individual genes in breast cancer dissemination have increased our knowledge. Although studies have revealed an important role for tumour proliferation‐related genes,^10^, ^11^, ^12^ the functional end result remains cell division. The latter is detectable for the examining pathologist as mitotic figures showing a typical appearance of chromosome sets. Assessment of mitotic figures, expressed as the mitotic activity index (MAI), is the oldest method of evaluating tumour proliferation and an important component of histological grade. The MAI has shown to be an important independent prognostic factor,^13^, ^14^ but its reproducibility remains limited.^15^, ^16^, ^17^

Tumour proliferation can also be determined immunohistochemically by staining for the proliferation‐related antigen Ki67. Several studies have demonstrated prognostic significance of assessing Ki67 in invasive breast cancer,^18^, ^19^ but variation in the methodology of this assay has limited its adoption in clinical practice.^20^, ^21^, ^22^, ^23^, ^24^

Phosphohistone H3 (PhH3) has been proposed as a novel proliferation marker. This protein is involved in chromatin condensation and decondensation, and is present in the active phases of the cell cycle (G_2_ to M transition). Unlike Ki67 assessment, PhH3 assessment is performed according to a standardised protocol, similar to that used for traditional mitosis counting. The contrast‐rich PhH3 staining enhances the recognition of mitotic figures, and the scoring resembles assessment of the MAI. PhH3 has been shown to have prognostic value in lymph node‐negative breast cancer patients,^25^ but studies regarding the reproducibility of PhH3 assessment in breast cancer are scarce. In the present study, we aimed to compare the interobserver agreement for assessment of the MAI, Ki67 and PhH3 in a cohort of oestrogen receptor (ER)‐positive breast cancer patients. Furthermore, the impact of replacing the MAI with PhH3 to determine histological grade was assessed.

## Materials and methods

### Patients

As part of a prospective observational multicentre study regarding the influence of the 70‐gene signature on adjuvant chemotherapy decision‐making in patients treated for ER+ early‐stage (i.e. absence of distant metastasis) invasive ductal breast cancer, tumour samples were obtained between 1 January 2013 and 31 December 2015. The study was approved by the medical ethics committee of the University Medical Centre Utrecht (12–450) and by the institutional review boards of participating centres. Patients enrolled in this study were asked for their consent to use their tumour samples for future research. The current side‐study was conducted according to the principles of Human Tissue and Medical Research: Code of conduct for responsible use (2011). For the present study, tissue samples of 159 patients were randomly retrieved from seven of the 31 participating centres.

### Clinicopathological Information

Clinicopathological data were obtained from the study database: patient age, tumour size, grade (based on nuclear polymorphism, tubular formation, and mitotic count), histological subtype, lymph node involvement, and ER, progesterone receptor (PR) and HER2 status.

### Pathological Examination

Pathological ER, PR and HER2 assessments had been routinely performed on all tumour samples (*n* = 159). Immunohistochemistry and fluorescence *in‐situ* hybridisation (FISH) were performed according to local standards at each institution. According to the Dutch guideline,^26^ positive ER or PR identification was defined as the presence of nuclear staining in ≥10% of breast cancer cells. Immunohistochemical expression of HER2 was scored as follows: 0 as <10% of tumour cells staining positively; 1+ as >10% of tumour cells staining positively, but no circumferential staining being present; 2+ as >10% of tumour cells showing weak or moderate circumferential staining; and 3+ as >10% of tumour cells showing strong circumferential staining. Scores of 0 and 1+ were considered to indicate a negative result, 2+ an equivocal result, and 3+ a positive result. HER2 2+ scores were re‐evaluated with FISH.

**Figure 1:**
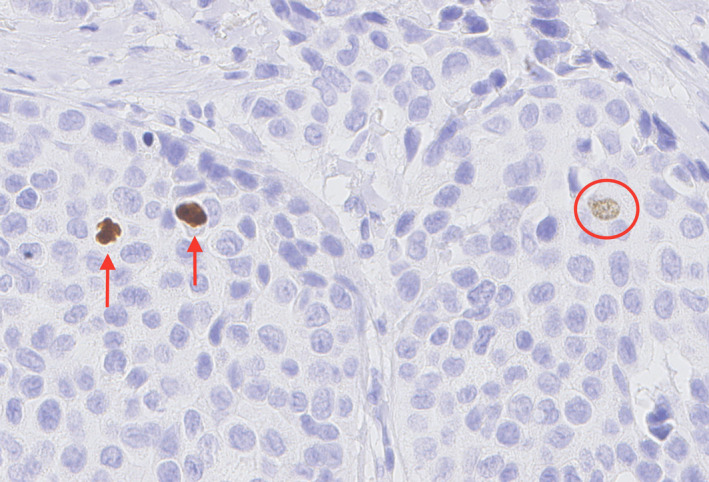
Microscopic image of phosphohistone H3 (PhH3) staining. True mitoses (arrows) are highlighted by the PhH3 immunostain. Intact nuclei with fine granular PhH3 staining (circle) were not counted, as these cells were regarded as not being in G_2_/M phase. [Colour figure can be viewed at wileyonlinelibrary.com]

Tissue samples were assessed for the MAI by three dedicated breast cancer pathologists, employed in different institutions, who were blinded to the clinicopathological data, according to the protocol guidelines of Van Diest and Baak.^27^ One pathologist (observer 2) assessed the MAI in 106 of 159 included patients, whereas the other two pathologists (observers 1 and 3) assessed the MAI in all 159 patients. MAI was categorised on the basis of the total number of mitotic figures in an area of 2 mm^2^, as follows: 0–7 = 1, 8–12 = 2, and ≥13 = 3. Whole tumour tissue sections of the 159 patients were immunohistochemically stained for PhH3 (clone BC37, 1:250; Biocare, Pacheco, CA, USA). The PhH3‐based mitotic count was scored by the same observers. As for traditional mitosis counting, the area of highest proliferation, preferably at the periphery of the tumour, was identified to assess the PhH3 mitotic count. PhH3‐positive objects, usually with mitosis morphology, were counted in an area of 2 mm^2^, whereas intact nuclei with fine granular PhH3 staining were not counted, as these cells were regarded as not being in the G_2_/M phase (Figure 1).^28^ The previously reported PhH3 threshold of 13 positive cells was used to discriminate between patients with a high or a low number of PhH3‐positive cells, as this cut‐off value was associated with 20‐year recurrence‐free survival rates for patients with distant metastases of 58% and 96%, respectively.^25^

In a non‐selected subset of 105 patients, tumour tissue was additionally stained for Ki67 in one laboratory (Mib‐1 antibody, ready‐to‐use; Dako, Glostrup, Denmark). Ki67 expression was assessed in 105 patients by observers 2 and 3, using the global scoring method. A cut‐off value of 20% of nuclei positively stained for Ki67 was used to discriminate between high‐proliferative and low‐proliferative tumours, as previously established.^29^

### Statistical Analyses

Data were analysed with r, Version 3.2.2. The intraclass correlation coefficient (ICC), determined with the two‐way random effects model for multiple raters [ICC with 95% confidence interval (CI)], was used to assess inter‐rater agreement for numerical variables (PhH3, Ki67 and MAI score on a continuous scale), and Cohen's *κ* was used to assess inter‐rater reliability for categorical variables (PhH3, Ki67 and the MAI categorised on the basis of the aforementioned thresholds). Furthermore, we created an alternative histological grade by replacing the MAI‐based mitotic count with the PhH3‐based mitotic count as follows: 1 point for a PhH3 mitotic number of ≤7 per 2 mm^2^; 2 points for a PhH3 mitotic number of 8–12 per 2 mm^2^; and 3 points for a PhH3 mitotic number of ≥13 per 2 mm^2^. This PhH3‐based histological grade of PhH3 was compared with the traditional MAI‐based grade by use of the chi‐square test. Two reasonable scales for the interpretation of the ICC and Cohen's *κ* are shown in Table S1.^30^

## Results

### Patients

In total, 159 early breast cancer patients with a median age of 57 years were included in this study. All patients had ER+ disease, 88% of patients had PR+ disease, and 98% of patients were HER2−. The majority of the patients had no axillary lymph node involvement (87%) (Table 1).

**Table 1:** Clinical and pathological characteristics of oestrogen receptor‐positive breast cancer patients included in the study (*n* = 159)

Characteristic	Value
Age (years), median (minimum–maximum)	57 (33–70)
Progesterone receptor status, *n* (%)	
Negative	19 (12)
Positive	140 (88)
HER2 status, *n* (%)	
Negative	156 (98)
Positive	2 (2)
Grade, *n* (%)	
1	26 (16)
2	106 (67)
3	27 (17)
Histological tumour type, *n* (%)	
Invasive ductal breast cancer	159 (100)
Unifocal tumour, *n* (%)	
No	9 (6)
Yes	150 (94)
Tumour diameter (mm), median (minimum–maximum)	15 (5–35)
T stage, *n* (%)	
T1	132 (83)
T2	27 (17)
N stage, *n* (%)	
N0	138 (87)
Nmi	13 (8)
N1a	6 (4)
Unknown	2 (1)
Type of surgery, *n* (%)	
Lumpectomy	134 (84)
Mastectomy	25 (16)

On the basis of the original pathology assessment, 16% of patients had low‐grade (I) cancers and 67% of patients had intermediate‐grade (II) tumours. For traditional mitosis counting, the median total number of mitotic figures were 2 [interquartile range (IQR) of 3], 3 (IQR of 6) and 5 (IQR of 8) for observers 1, 2 and 3, respectively, resulting in an MAI score of 1 in 84%, 70% and 60% of patients.

The median total PhH3 scores were 10 (IQR of 18), 8 (IQR of 12) and 9 (IQR of 16) for observers 1, 2, and 3, respectively. The percentages of low‐proliferative tumours based on the PhH3 mitotic count (<13 points per 2 mm^2^) were 60% (observer 1), 62% (observer 2), and 63% (observer 3) (Table 2). Median numbers of total nuclei positively stained for Ki67 were 5 (IQR of 5) and 2 (IQR of 3) for observers 2 and 3, respectively. The percentages of low‐proliferative tumours based on the Ki67 score (<20% of positively stained nuclei) were 81% and 89%, respectively (Table 2).

**Table 2:** Mitotic activity index, phosphohistone H3 (PhH3) scores and Ki67 percentages assessed by three different breast cancer pathologists

	Observer 1 (*n* = 159)	Observer 2 (*n* = 106)	Observer 3 (*n* = 159)
Mitotic activity index			
1 (0–7 mitotic figures) per 2 mm^2^	134	74	94
2 (8–12 mitotic figures) per 2 mm^2^	13	19	28
3 (≥13 mitotic figures) per 2 mm^2^	12	13	37
PhH3 score			
<13 positively stained cells per 2 mm^2^	95	66	100
≥13 positively stained cells per 2 mm^2^	64	40	59
Ki67 percentage			
<20% positively stained cells	–	85	93
≥20% positively stained cells	–	20	12
Not assessed	159	1	54

PhH3, Phosphohistone H3.

### Agreement of Continuous PhH3, MAI and Ki67 Scores

**Figure 2:**
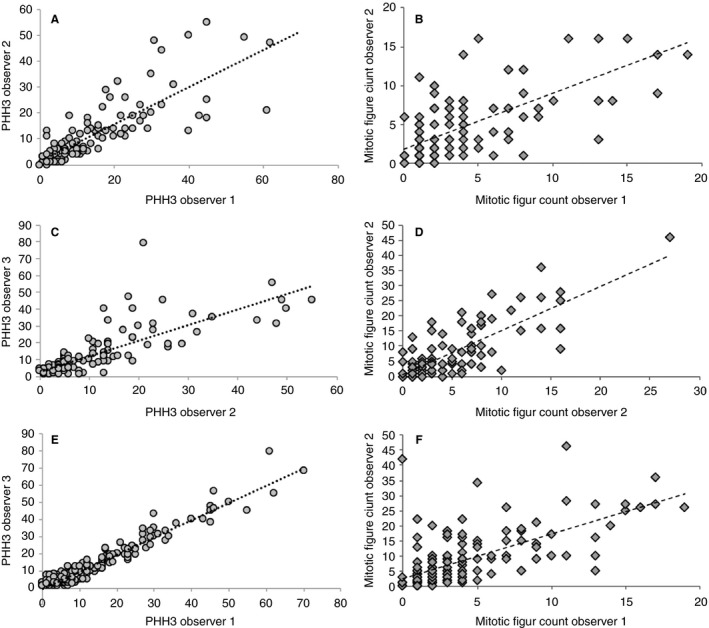
**A**–**C**, Interobserver agreement of the total phosphohistone H3 (PhH3) score for observers 1 and 2 (**A**), observers 2 and 3 (**B**), and observers 1 and 3 (**C**). **D**–**F**, Total mitotic figure count assessed in an area of 2 mm^2^ by observers 1 and 2 (**D**), observers 2 and 3 (**E**), and observers 1 and 3 (**F**). **A**, PhH3 intraclass correlation coefficient (ICC) score of 0.79 [95% confidence interval (CI) 0.67–0.86]. **B**, PhH3 ICC score of 0.76 (95% CI 0.72–0.86). **C**, PhH3 ICC score of 0.97 (95% CI 0.96–0.98). **D**, Mitotic count ICC of 0.62 (95% CI 0.48–0.72). **E**, Mitotic count ICC of 0.61 (95% CI 0.39–0.75). **F**, Mitotic count ICC of 0.42 (95% CI 0.16–0.60).

The ICCs of the PhH3 mitotic count on a continuous scale for observer 1 versus 2, observer 1 versus 3 and observer 2 versus 3 were 0.79 (95% CI 0.67–0.86), 0.97 (95% CI 0.96–0.98) and 0.76 (95% CI 0.72–0.86), respectively (Figure 2A–C). Interobserver agreement for PhH3 among all three pathologists reflected almost perfect agreement (ICC of 0.86, 95% CI 0.80–0.89). The ICCs for the total mitotic figure count were lower than those for PhH3: 0.62, 95% CI 0.48–0.72 (observer 1 versus observer 2), 0.41, 95% CI 0.16–0.60 (observer 1 versus observer 3), and 0.61, 95% CI 0.39–0.75 (observer 2 versus observer 3) (Figure 2D–F). The ICC for the total mitotic figure count among all three pathologists was 0.57 (95% CI 0.41–0.69). The ICC for Ki67 for the two pathologists who assessed Ki67 was 0.64 (95% CI 0.39–0.78) (observer 1 versus observer 2).

### Agreement of Categorical PhH3, MAI and Ki67 Scores

The *κ* scores for the categorical PhH3 score (*κ* = 0.78 for observer 1 versus observer 2, *κ* = 0.80 for observer 1 versus observer 3, *κ* = 0.68 for observer 2 versus observer 3; Table S2) reflected substantial agreement between the three observers. Interobserver agreement for Ki67 and the MAI was only fair to moderate: Ki6, *κ* = 0.55 (observer 1 versus observer 2); and MAI, *κ* = 0.38 (observer 1 versus observer 2), *κ* = 0.26 (observer 1 versus observer 3), and *κ* = 0.52 (observer 2 versus observer 3), respectively (Tables S3 and S4).

### Assessment of Histological Grade Based on the MAI Versus Grade Based on PhH3

When PhH3 was used in the modified BR Nottingham grading score instead of the MAI, interobserver agreement in determining histological grade improved (MAI, *κ* = 0.43, *κ* = 0.35, and *κ* = 0.32; PHH3, *κ* = 0.52, *κ* = 0.48, and *κ* = 0.52). At the same time, when the grading score was re‐evaluated on the basis of PhH3 assessment, it shifted from grade I to grade II in 8% (observer 1), 12% (observer 2) and 4% (observer 3) of the patients, and from grade II to III in 27% (observer 1), 18% (observer 2) and 14% (observer 3) of the patients (*P* < 0.001) (Table 3). Among all three observers, there were a few patients who were downgraded from grade II to grade I (*n* = 1, *n* = 2 and *n* = 3 for observers 1, 2 and 3, respectively) or downgraded from grade III to grade II (*n* = 1, *n* = 2 and *n* = 6 for observers 1, 2, and 3, respectively) (Table 3). The majority of the patients who were upgraded from grade II to grade III had a PhH3 score of ≥13 (86%, 95% and 69% for observers 1, 2 and 3, respectively), whereas a substantial proportion in whom the histological grade was shifted from grade I to II had a PhH3 score of <13 (31%, 43% and 83% for observers 1, 2 and 3, respectively).

**Table 3:** Impact of replacing the mitotic activity index with phosphohistone H3 on the modified Bloom–Richardson Nottingham grade score

Change in histological grading score	Observer 1 (%)	Observer 2 (%)	Observer 3 (%)
Upgraded from grade I to grade II	8	12	4
Upgraded from grade I to grade III	–	1	–
Upgraded from grade II to grade III	27	18	14
Downgraded from grade II to grade I	1	2	2
Downgraded from grade III to grade II	1	2	4

## Discussion

In this study, the reproducibility of three different proliferation‐related variables that contribute to the assessment of tumour grade was compared in patients with luminal‐type breast cancer. Our results demonstrate that PhH3‐based mitotic counting provides a more reproducible means for observing tumour proliferation in ER+ early breast cancers than MAI or Ki67 assessment. Incorporating PhH3 as an alternative to the traditional MAI in the BR Nottingham grading system would decrease the variation in histological grading, but would increase the proportion of cancers that would be considered to be high‐grade tumours.

Assessment of mitotic activity is routinely performed as part of determining histological tumour grade, and has been established as an independent prognostic factor.^31^, ^32^, ^33^, ^34^ The reproducibility of the MAI is limited.^15^, ^16^, ^17^ This may in part be attributable to a lack of strict protocols, and to difficulties in selecting the mitotically most active area,^16^, ^17^ but it may also result from the coexistence of cells that mimic mitosis, such as apoptotic and necrotic cells, especially in cases of poor fixation.^35^ Optimal assessment of mitotic activity requires the experience of trained pathologists and dedication, as this may take ~10 min.^36^

PhH3 showed better interobserver agreement in the present study than did the MAI, supported by higher ICC and Cohen's *κ* scores. PhH3 is a proliferation marker that is specific for mitosis, as it is expressed from the late G_2_ phase to M transition, and rapidly degrades on entry into the G_1_ phase.^37^ Therefore, PhH3 labelling has been reported to closely correlate with mitotic figure detection on standard haematoxylin and eosin (H&E)‐stained sections.^38^, ^39^ As compared with the MAI, PhH3 is relatively easy to assess, as its bright staining offers easy visualisation of mitotic figures by morphology, resulting in a high accuracy of detection. The results of our study showed that PhH3 revealed higher numbers of mitotic cells than did H&E staining, which is in line with previous literature.^28^, ^40^ This difference in sensitivity may be explained by the fact that prophase figures are not well recognised with regular H&E stains, but can be easily identified in PhH3‐stained specimens.^28^ Because of the sharp contrast with non‐stained elements, PhH3 allows rapid detection of the mitotically most active area.^40^ A previous study demonstrated that PhH3 staining was particularly useful in detecting mitotic cells in high‐grade cancers with dense cellularity and with numerous apoptotic and necrotic cells.^28^ In addition, PhH3 assessment may serve as a better means to assess proliferative activity in core needle biopsies, as PhH3 labelling was found to be more accurate at identifying mitotic figures than routine H&E staining.^41^ In the light of these advantages, it is conceivable that PhH3 staining results in a higher accuracy of mitotic figure detection, even in specimens with poor fixation, or specimens that contain dense, distorted tumour infiltrate or crush artefacts. Then again, others have shown that antigenicity for PhH3 can be lost if tissue is not immediately fixed after sampling.^42^ Hence, fixation delay should be kept as short as possible.

In addition to the conventional factors, immunohistochemical assessment of the proportion of cells staining for the nuclear antigen Ki67 is used for determination of tumour proliferation. Many studies have demonstrated the prognostic value of Ki67.^43^ However, the clinical utility of this marker has been disputed because of poor reproducibility, which is also reflected by the results of the present study. Flaws in Ki67 assessment are attributed to a lack of scoring consensus among experts and an undefined cut‐off point for clinical decision‐making. In an effort to harmonise the analytical methodology of Ki67, the International Ki67 Breast Cancer Working Group proposed a set of guidelines for the analysis and reporting of Ki67.^44^ However, even after standardisation, the assessment of Ki67 among some of the world's most experienced laboratories turned out to be poor.^45^ Although interlaboratory variability in staining methods contributed to differences in Ki67 scoring, the working group also observed substantial discrepancies in Ki67 interpretation when the staining was performed centrally. These results are in line with those of another study reporting high interobserver variability in Ki67 assessment among 15 pathologists.^46^ The Ki67 working group stated that ‘unless an individual pathology laboratory has demonstrated that its staining and scoring methodology, including cut‐off determination, meet the highest level of evidence for clinical utility, clinicals should use Ki67 results with caution’.^45^, ^47^

As PhH3 assessment is also based on immunohistochemistry, one may wonder to what extent PhH3 assessment suffers from similar limitations. In contrast to the variability in Ki67 scoring methods, PhH3 assessment is performed according to a standardised protocol similar to that used for traditional mitosis counting. Furthermore, PhH3‐positive cells can be unambiguously identified, even at low‐power magnification and by inexperienced observers.^40^ Finally, there is less debate regarding cut‐off values for PhH3 assessment.

In the present study, the use of PhH3 instead of the MAI to determine the modified BR histological grade resulted in the histological grade being upgraded in 14–27% of cases. This increase in the proportion of patients with high‐grade tumours is in line with other studies.^25^, ^28^, ^38^, ^48^ PhH3 was shown to have independent prognostic value, which exceeded the prognostic value of the MAI [hazard ratio (HR) of 9.6 versus HR of 3.6].^49^ These findings support the concept of replacing the MAI with PhH3 in order to improve the prognostic value of histological grading through better identification of mitotic figures. At the same time, PhH3‐based mitotic indices should be evaluated in larger studies before their use in clinical practice can be recommended.

To our knowledge, this study has provided a unique comparison between the reproducibility of traditional proliferation markers and that of the novel proliferation marker PhH3. Interobserver agreement was reliable, as the pathology examination was performed by three dedicated breast cancer pathologists, working in different institutions. It is important to note that we performed this study in a selection of ER+ cancers, and this should be taken into consideration when the results are interpreted. However, optimisation of the assessment of tumour proliferation is especially needed in this subset of patients, as the patient group was a selected group in whom genomic profiling was undertaken to decide on adjuvant chemotherapy. It is important to note that the prognostic value of the different proliferation markers was not addressed in the present study, as follow‐up data were not available, and the follow‐up period would have been too short. In due course, outcome data will become available, and these will enable us to also further evaluate PhH3 assessment in terms of prognostication. We also aim to explore deep‐learning algorithms to automatically identify PhH3‐positive objects, as has successfully been performed before for mitoses in H&E‐stained and PhH3‐stained sections.^50^, ^51^

In conclusion, our results demonstrate that PhH3 is a more reproducible proliferation marker in breast cancer than are the MAI and Ki67. The association between PhH3 and outcome, and the potential increase in the proportion of high‐grade cancers when PhH3 is used, need to be further addressed.

## Conflicts of interest

The authors declare that there are no conflicts of interest. No funding was received for this study.

## Author contributions

All persons listed as authors were actively involved in one or more key aspects of the reported study. J. E. C. van Steenhoven: conception and design, analysis and interpretation of data, drafting of the article, and final approval. A. Kuijer: conception and design, acquisition of data, analysis and interpretation of data, critical revision, and final approval. R. Kornegoor: interpretation of data, critical revision, and final approval. A. M. van Leeuwen: acquisition of data, critical revision, and final approval. J. van Gorp: acquisition of data, interpretation of data, critical revision, and final approval. T. van Dalen: conception and design, interpretation of data, drafting of the article, and final approval. P. J. van Diest: acquisition of data, interpretation of data, critical revision, and final approval.

## Supporting information


**Table S1.** Interpretation of intraclass correlation coefficient (ICC) and Cohen's *κ* score.
**Table S2.** Concordance of PhH3 scored by three different breast cancer pathologists.
**Table S3.** Concordance of MAI classes scored by three different breast cancer pathologists.
**Table S4.** Concordance of Ki67 scored by two different breast cancer pathologists.
