# Supplementary material for: Assessment of tumour proliferation by use of the mitotic activity index, and Ki67 and phosphohistone H3 expression, in early‐stage luminal breast cancer
Source: Histopathology. 2020 Aug 30;77(4):579–87. doi: 10.1111/his.14185 (PMC7539961; doi:10.1111/his.14185)
Supplement: Supplementary file 1 — Table S1. Interpretation of intraclass correlation coefficient (ICC) and Cohen's κ score. Table S2. Concordance of PhH3 scored by three different breast cancer pathologists. Table S3. Concordance of MAI classes scored by three different breast cancer pathologists. Table S4. Concordance of Ki67 scored by two different breast cancer pathologists. [file HIS-77-579-s001.docx]

**Supplementary table 1.** Interpretation of Intraclass Correlation Coefficient (ICC) and Cohen’s Kappa score.

| ICC | Interpretation of ICC |
| --- | --- |
| <0.5 | Poor agreement |
| 0.5-0.74 | Moderate agreement |
| 0.75-0.9 | Good agreement |
| >0.9 | Excellent agreement |
|  |  |
| Kappa | **Interpretation of Cohen’s Kappa score** |
| < 0 | Poor agreement |
| 0.01-0.20 | Slight agreement |
| 0.21-0.40 | Fair agreement |
| 0.41-0.60 | Moderate agreement |
| 0.61-0.80 | Substantial agreement |
| 0.81-1.00 | Almost perfect agreement |
|  |  |

**Supplementary table 2.** Concordance of PhH3 scored by three different breast cancer pathologists.

|  | Obs. 2 | | | Obs. 3 | | |  | |  | | Obs. 3 | |
| --- | --- | --- | --- | --- | --- | --- | --- | --- | --- | --- | --- | --- |
| Obs. 1 | **PhH3<13** | **PhH3 ≥13** | | **PhH3<13** | **PhH3≥13** | | |  | | **Obs. 2** | **PhH3<13** | **PhH3≥13** |
| PhH3<13 | 58 | | 3 | 90 | | 5 |  | | **PhH3<13** | | 59 | 7 |
| PhH3 ≥13 | 8 | | 37 | 10 | | 54 |  | | **PhH3 ≥13** | | 9 | 31 |
| The overall concordance of PhH3 for observer 1 vs. 2, 1 vs. 3 and 2 vs. 3 were 90% (κ 0.78), 91% (κ 0.80) and 85% (κ 0.68), respectively. Abbreviations: obs, observer, PhH3, phosphohistone H3 | | | | | | | | | | | | |

**Supplementary table 3.** Concordance of MAI classes scored by three different breast cancer pathologists.

|  | Obs. 2 | | | Obs. 3 | | |  |  | Obs. 3 | | |
| --- | --- | --- | --- | --- | --- | --- | --- | --- | --- | --- | --- |
| Obs. 1 | **MAI**  **0-7** | **MAI**  **8-12** | **MAI**  ≥**13** | **MAI**  **0-7** | **MAI**  **8-12** | **MAI ≥13** |  | **Obs. 2** | **MAI**  **0-7** | **MAI**  **8-12** | **MAI**  **≥13** |
| MAI  0-7 | 71 | 11 | 6 | 92 | 23 | 19 |  | **MAI**  **0-7** | 62 | 8 | 4 |
| MAI  8-12 | 1 | 5 | 3 | 1 | 3 | 9 |  | **MAI**  **8-12** | 2 | 8 | 9 |
| MAI  ≥13 | 2 | 3 | 4 | 1 | 2 | 9 |  | **MAI**  **≥13** | 2 | 1 | 10 |

The overall concordance of MAI for observer 1 vs. 2, observer 1 vs. 3 and observer 2 vs. 3 were 75% κ 0.38 95% CI 0.10-0.42, 65% κ 0.26 95% CI 0.10-0.42 and 75% k 0.52 95% CI 0.36-0.68 respectively. Abbreviations: obs, observer, MAI, Mitotic Activity Index

**Supplementary table 4.** Concordance of Ki67 scored by two different breast cancer pathologists.

|  | Obs. 3 |  |
| --- | --- | --- |
| Obs. 2 | **Ki67 <20%** | **Ki67 ≥20%** |
| Ki67 <20% | 83 | 2 |
| Ki67 ≥20% | 10 | 10 |

The overall concordance of Ki67 for observer 1 vs. 2 was 88% k 0.52 95% CI 0.30-0.74
